# Grain Boundary Evolution of Cellular Nanostructured Sm-Co Permanent Magnets

**DOI:** 10.3390/ma14185179

**Published:** 2021-09-09

**Authors:** Wei Zhang, Hongyu Chen, Xin Song, Tianyu Ma

**Affiliations:** 1 College of Mechanical and Electrical Engineering, Henan Agricultural University, Zhengzhou 450002, China; zwz04@163.com; 2 Frontier Institute of Science and Technology, Xi’an Jiaotong University, Xi’an 710054, China; lovehuacai@stu.xjtu.edu.cn (H.C.); xin13525689435@stu.xjtu.edu.cn (X.S.)

**Keywords:** permanent magnets, cellular nanostructure, grain boundary, phase transformation, precipitates

## Abstract

Grain boundaries are thought to be the primary demagnetization sites of precipitate-hardening 2:17-type Sm-Co-Fe-Cu-Zr permanent magnets with a unique cellular nanostructure, leading to a poor squareness factor as well as a much lower than ideal energy product. In this work, we investigated the grain boundary microstructure evolution of a model magnet Sm_25_Co_46.9_Fe_19.5_Cu_5.6_Zr_3.0_ (wt. %) during the aging process. The transmission electron microscopy (TEM) investigations showed that the grain boundary region contains undecomposed 2:17H, partially ordered 2:17R, 1:5H nano-precipitates, and a Sm_n+1_Co_5n−1_ (n = 2, 1:3R; n = 3, 2:7R; n = 4, 5:19R) phase mixture at the solution-treated state. After short-term aging, further decomposition of 2:17H occurs, characterized by the gradual ordering of 2:17R, the precipitation of the 1:5H phase, and the gradual growth of Sm_n+1_Co_5n−__1_ compounds. Due to the lack of a defect-aggregated cell boundary near the grain boundary, the 1:5H precipitates are constrained between the 2:17R and the Sm_n+1_Co_5n−1_ nano-sheets. When further aging the magnet, the grain boundary 1:5H precipitates transform into Sm_n+1_Co_5n−1_ compounds. As the Sm_n+1_Co_5n−1_ compounds are magnetically softer than the 1:5H precipitates, the grain boundaries then act as the primary demagnetization sites. Our work adds important insights toward the understanding of the grain boundary effect of 2:17-type Sm-Co-Fe-Cu-Zr magnets.

## 1. Introduction

The alloys of 2:17-type Sm-Co-Fe-Cu-Zr are the strongest high-temperature permanent magnets and have been widely applied in advanced industries such as large-efficiency motors, magnetic bearings, and the momentum wheel of satellite communications because they can maintain a high maximum energy product ((*BH*)_max_) and high coercivity (*H*_cj_) at elevated temperatures [1,2,3,4,5,6,7]. The hard magnetism of Sm-Co-Fe-Cu-Zr magnets is pinning-controlled and is closely related to the unique cellular nanostructure of 2:17R nanocells, 1:5H cell boundary precipitates, and 1:3R lamellar precipitates, formed by aging the solution-treated precursors with micron-sized grains (several tens of microns) [1,2]. However, the squareness factor of Sm-Co-Fe-Cu-Zr magnets is lower than that of nucleation-controlled Nd-Fe-B magnets [8,9,10,11], thus leading to a much lower than ideal (*BH*)_max_, the key figure of merit of permanent magnets. Therefore, understanding the microstructural origin of the poor squareness factor in pinning-controlled Sm-Co-Fe-Cu-Zr magnets has been one of the most important issues.

It has been stated that Cu-enriched 1:5H cell boundary precipitates can provide a strong pinning force to hinder the motion of magnetic domain walls (DWs), giving rise to a large coercivity [3,5,12,13,14,15,16,17,18,19]. An ideal microstructure configuration for achieving strong hard magnetic properties is that all the 2:17R nanocells should be surrounded by continuous 1:5H precipitates. However, the magnets usually contain various types of microstructural deficiencies, which weaken the pinning force against DW motions locally and lead to an inhomogeneous demagnetization process as well as a poor squareness factor. These microstructural deficiencies include: (i) the intersections between 1:5H precipitates and the co-existing 1:3R precipitates [14], (ii) the cell edges that usually contain stacking faults (SFs) or the 2:17R’ intermediate phase [20], (iii) the defect-aggregated cell boundaries (DACBs) free of the 1:5H phase [21], and (iv) the grain boundaries with sparser 1:5H cell boundary precipitates (i.e., larger cells) than the grain interiors and phase mixture of Zr-stabilized Sm_n+1_Co_5n−1_ (n = 2, 1:3R; n = 3, 2:7R; n = 4, 5:19R) precipitates that are magnetically softer than the 1:5H precipitates [15,22,23,24,25,26,27]. Among them, the grain boundaries have been thought to be the primary demagnetization sites [5,28]. Unlike the first three types of deficiencies that are formed by the decomposition from 1:7H or 2:17H into the phase mixture of the 2:17R/2:17R’ phase, 1:5H, and 1:3R precipitates, the formation of the last type—grain boundary deficiencies—is more complex especially for the Fe-rich Sm-Co-Fe-Cu-Zr magnets with a large theoretical (*BH*)_max_. Firstly, there is an apparent microstructure difference between the grain boundaries and grain interiors even at the solution-treated state including a higher 2:17R ordering degree, fewer DACBs to be occupied by the 1:5H precipitates during the subsequent aging process, and even Zr-stabilized Sm_n+1_Co_5n−1_ primary precipitates [23,24,29]. Secondly, the kinetics either for recrystallization or precipitation are distinct between the grain boundaries and grain interiors; the concurrently happening and strongly interacted recrystallization (formation and growth of the nanocells) and precipitation in the Sm-Co magnets make the decomposition kinetics more complex at the grain boundaries during the subsequent aging process. Thirdly, during aging, there is also the growth of Sm_n+1_Co_5n−1_ precipitates at the grain boundaries [24], which may exert a strong influence on the growth of recrystallized cells and the precipitation of 1:5H precipitates. Consequently, it is necessary to investigate how the grain boundary microstructure evolves during the aging process, i.e., how the concurrently happening recrystallization, precipitation of 1:5H precipitates, and growth of Sm_n+1_Co_5n−__1_ precipitates interact with each other.

In this work, we performed a detailed high-resolution transmission electron microscopy (HR-TEM, JEOL, Tokyo, Japan) investigation on the Sm_25_Co_46.9_Fe_19.5_Cu_5.6_Zr_3.0_ (wt. %) magnets with a higher magnetic performance identified from previous works [24,30]. The microstructure evolution at the grain boundaries during the aging process was revealed. The HR-TEM images clearly showed that both the recrystallized 2:17R nanocells and Sm_n+1_Co_5n−__1_ precipitates grew quickly whereas sparse 1:5H cell boundary precipitates were formed during the aging process, which led to a poor squareness factor and a maximum energy product. In addition, the interaction among the concurrently happening recrystallization, precipitation of the 1:5H phase, and growth of Sm_n+1_Co_5n−__1_ precipitates is also discussed.

## 2. Materials and Methods

Magnets with a nominal composition of Sm_25_Co_46.9_Fe_19.5_Cu_5.6_Zr_3.0_ (wt. %) were fabricated using the powder metallurgy method under the protection of high-purity argon. The powders, with an average size of ~5 μm, were pressed under ~150 MPa in a magnetic field higher than 10 kOe followed by isostatic compaction under ~200 MPa. The green compacts were sintered for ~3 h at ~1200 °C followed by homogenization for 4 h at ~1170 °C. The magnets were then quenched by a rapid high-purity argon flow to obtain the solution-treated precursors. Subsequently, a few of the solution-treated precursors were aged for ~10 h at 810 °C, then slowly cooled to 400 °C at 0.7 °C/min and aged again for 1 h to obtain the final magnets. To reveal the microstructure evolution at the grain boundaries during the aging process, a few of the solution-treated precursors were also aged for 0.5 h at 810 °C and quenched.

The initial magnetization curves of the thermally demagnetized samples and the magnetization hysteresis loops were measured up to 70 kOe using a physical property measurement system magnetometer (Quantum Design, Inc., San Diego, CA, USA). The average structure of the powder samples was characterized using an X-ray diffractometer (XRD) with Cu *K*_α_ radiation (Rigaku Corporation, Tokyo, Japan). Detailed microstructures of the samples were characterized using a JEM-2100F TEM (JEOL, Tokyo, Japan) with a beam voltage of 200 kV. The TEM foil samples were prepared by standard grinding, polishing, dimpling, and ion thinning. The selected area electron diffraction (SAED) patterns were taken by tilting the selected grain along the [100]2:_17R_ zone axis (ZA). The HR-TEM images were analyzed using DigitalMicrograph software (Gatan Inc., Pleasanton, CA, USA).

## 3. Results

**Figure 1:**
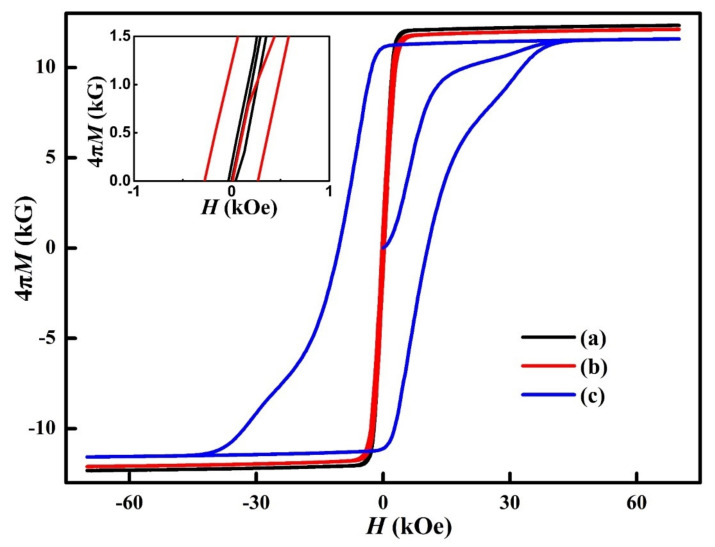
Initial magnetization curves and magnetization hysteresis loops of the samples at different states: solution-treated (**a**), aged for 0.5 h at 810 °C (**b**), and the final magnet (**c**). The inset is an enlarged view of the curves for the solution-treated sample and the 0.5 h-aged sample.

Figure 1 shows the room temperature initial magnetization curves and magnetization hysteresis loops of the Sm_25_Co_46.9_Fe_19.5_Cu_5.6_Zr_3.0_ magnets at different states. The derived magnetic properties for the final magnet were (*BH*)_max_ = 21.31 MGOe, *B*_r_ = 11.61 kG, *H*_cj_ = 11.52 kOe, and squareness factor = 29.27%. The squareness factor was evaluated by *H*_k_/*H*_cj_, where *H*_k_ is the knee point field of the demagnetization curve at which 80% *J*_r_ is retained. The obtained squareness factor was much lower than that of the nucleation-controlled Nd_2_Fe_14_B-type sintered magnets, which is usually above 90% [8]. This was due to the fact that the hard magnetism in the 2:17-type Sm-Co-Fe-Cu-Zr magnets was dominated by domain wall pinning (as seen from the initial magnetization curve of the final magnet). We also measured the magnetization hysteresis loops of the solution-treated and the 0.5 h-aged samples, both of which showed a very small coercivity. It indicated that the 1:5H cell boundary precipitates, which play an essential role on the coercivity, had a very small fraction at the solution-treated state or after aging for 0.5 h.

**Figure 2:**
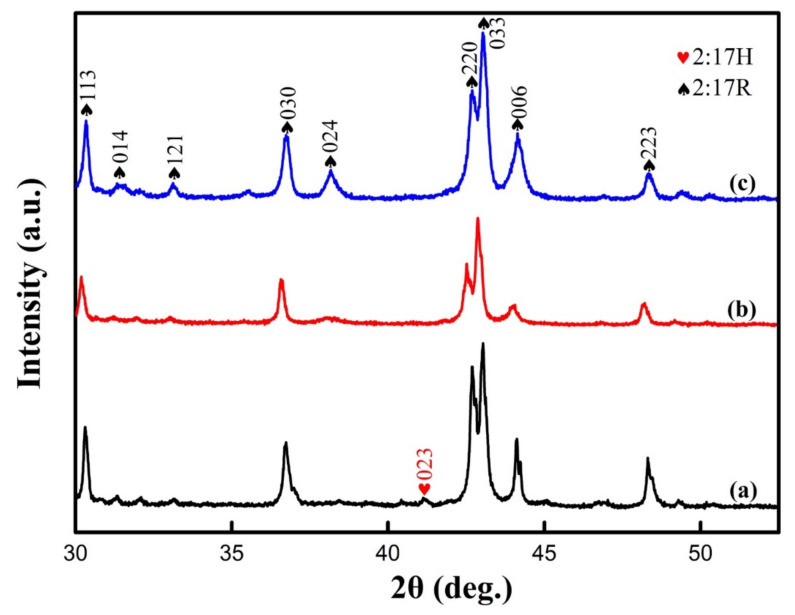
Powder XRD patterns of Sm_25_Co_46.9_Fe_19.5_Cu_5.6_Zr_3.0_ magnets: solution-treated (**a**), aged for 0.5 h at 810 °C (**b**), and the final magnet (**c**).

Figure 2 shows the powder XRD patterns of the magnets. The fundamental reflections of all the samples could be contributed from the phases reported in the 2:17-type Sm-Co magnets including 1:5H, 1:7H, 2:17H, 2:17R, 2:17R’, and Sm_n+1_Co_5n−1_ phases [25,31,32,33,34,35,36]. In Figure 2a, a very weak peak of {023} was present at 2*θ* of ~40.3° for the solution-treated sample, which is the characteristic peak of 2:17H [35]. In addition, weak superlattice reflections {014} and {121} unique to the 2:17R phase were also observed at low Bragg angles. These indicated that either the solution-treated sample contained a small fraction of 2:17H and 2:17R phases or the 2:17H- and 2:17R-type ordering degree of this sample was very low. After aging for 0.5 h, as shown in Figure 2b, the superlattice reflection {023} of the 2:17H phase disappeared, indicating that the decomposition of 2:17H had occurred. In addition, judging from the weak {024} superlattice reflection unique to the 2:17R phase, the 0.5 h–aged sample exhibited partial 2:17R ordering as the final state with a well-ordered 2:17R phase produced a much stronger {024} superlattice reflection, as shown in Figure 2c. The gradually strengthened superlattice reflection {024} with the aging time then suggested that aging effectively increased the ordering degree or mass fraction of the 2:17R phase.

**Figure 3:**
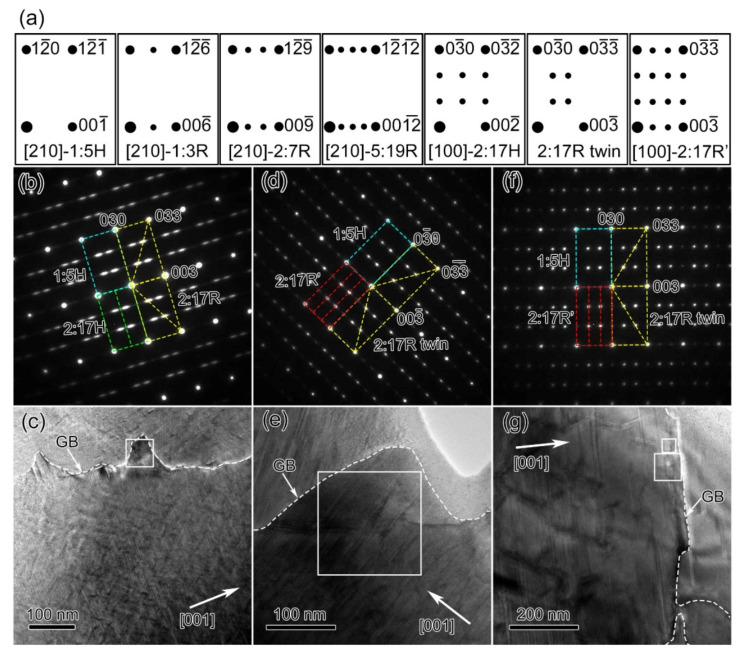
Single-crystal electron diffraction patterns for 1:5H, 1:3R, 2:7R, 5:19R, 2:17H, 2:17R twin variants, and the 2:17R’ phases (**a**). Selected area electron diffraction (SAED) patterns and bright-field TEM images for Sm_25_Co_46.9_Fe_19.5_Cu_5.6_Zr_3.0_ magnets at different states: solution-treated (**b**,**c**), aged for 0.5 h at 810 °C (**d**,**e**), and the final magnet (**f**,**g**).

Prior to presenting the TEM characterization results, it should be noted that the 2:17R phase exhibits a nanotwin structure in 2:17-type Sm-Co-Fe-Cu-Zr magnets [37,38]. The 1:5H precipitates usually occupy the {011}_2:17R_ pyramidal cell boundaries and the 1:3R lamellar precipitates usually occupy the {001} basal planes across the cells and cell boundaries [32,33,35]. Recent work has revealed that there is also a 2:17R’ phase, the intermediate phase of 2:17R, having one faulted basal layer in the 2:17R lattice, which is usually observed at the cell edges [20]. In Fe-rich magnets, 1:3R, 2:7R, 5:19R, or their mixtures have also been observed at the grain boundaries [12,13,16,37,39]. The primitive orientation relationship between 2:17R and 1:5H or Sm_n+1_Co_5n−1_ is (003)_2:17R_//(001)_1:5H/(n+1):(5n−1)_ and [100]2:17R//[210]1:5H/_(n+1):(5n−1)_, with the lattice parameters *a*_2:17R_ slightly smaller than $\sqrt{3}$*a*_1:5H/(n+1):(5n−1)_, *c*_2:17R_ slightly larger than 3*c*_1:5H_, *a*_(n+1):(5n−1__)_ nearly equivalent to *a*_1:5H_, and *c*_(n+1):(5n−1__)_ slightly smaller than 3n*c*_(n+1):(5n−1__)_ [2,40,41]. As shown in Figure 3a, the Sm_n+1_Co_5n−1_ precipitates produced extra superlattice reflections along the [001]* axis in the [100]2:17R SAED pattern. In addition to the ones overlapped with the superlattice reflections of the 2:17R twins, the 2:17R’ phase also produced extra superlattice reflections at 1/3 and 2/3 positions along both [001]* and [010]* directions. These features then allowed the distinguishing of all the above-mentioned phases along the specific [100]2:17R ZA.

**Figure 4:**
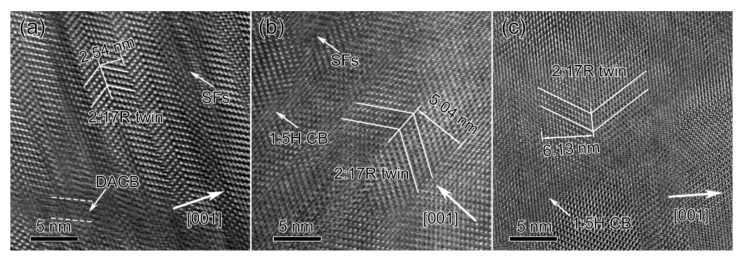
HR-TEM images of the grain interiors for Sm_25_Co_46.9_Fe_19.5_Cu_5.6_Zr_3.0_ magnets. Solution-treated (**a**), aged for 0.5 h at 810 °C (**b**), and the final magnet (**c**).

Figure 3b–g shows the SAED patterns and bright-field TEM images along the [100]2:17R ZA of all samples. From the SAED pattern of the solution-treated sample in Figure 3b, the elongated superlattice reflections {011}, {012}, {021}, and {022} were attributed to the partially ordered 2:17R phase. There were also weak diffuse streaks along the [001]* axis well covering the {011}*_2:17H_ and {021}*_2:17H_ positions, indicating that this sample also contained a short-range ordered 2:17H phase. After aging, as shown in Figure 3d,f, the superlattice reflections {0$\bar{11}$}* and {0$\bar{21}$}* of 2:17H phase disappeared and there was a disordered rhombohedral phase (2:17R’), producing 1/3 and 2/3 superlattice reflections along the [001]*2:17R and [010]*2:17R directions. Note that the 2:17R’ phase had one faulting layer in the 2:17R twins with an ACBA or ABCA basal stacking sequence [23,33,34,42], thus appearing as SFs. The diffuse steaks along [001]*2_:17R_ showed that there were basal SFs and/or Sm_n+1_Co_5n−1_ precipitates as Sm_n+1_Co_5n−1_ precipitates also generate diffuse streaks. In addition, the superlattice reflections of the 2:17R phase became sharper, indicating that the 2:17R phase became more ordered during the aging process, which was consistent with the XRD results in Figure 2. The gradual 2:17R ordering could be clearly seen from the HR-TEM images (shown in Figure 4) where the 2:17R variants gradually became wider from the solution-treated state to the final state.

In the bright-field TEM image (Figure 3c), a fine cellular nanostructure (the average cell size was estimated by the distance between the parallel pyramidal cell boundaries) had already been formed in the solution-treated state where the pyramidal cell boundaries (subgrain boundaries) were in dark contrast. The HR-TEM image shown in Figure 4a revealed that the cell boundary was free of 1:5H precipitates but was aggregated with defects. Therefore, the majority of the dark contrasts observed in Figure 3c should belong to DACBs, which act as the nucleation sites of 1:5H precipitates [18]. In the grain interiors, the average cell size was ~29.1 nm for the solution-treated magnets. Note that the cells were coarser nearer the grain boundaries than those at the grain interiors, i.e., the cell boundaries were sparser nearer the grain boundaries. This indicated that the nucleation sites were sparser nearer the grain boundaries when compared with the grain interiors. After aging, the cells grew together with the precipitations of the 1:5H and 1:3R phases, as shown in Figure 3d,f and Figure 4b,c. The average cell size was ~43.6 nm for the 0.5 h-aged magnet and ~135.2 nm for the final magnet. The 1:5H precipitates were observed at the cell boundaries in the 0.5 h-aged magnet (Figure 4b) and the final magnet (Figure 4c). Note that the decomposition also evolved into a gradual reduction in stacking faults (SFs) within the cells, which can also be clearly seen in Figure 4. It should be addressed that, after aging, the cell boundaries were still sparser near the grain boundaries than those at the grain interiors, i.e., the cells grew faster nearer the grain boundaries than those at the grain interior. More importantly, nano-sheets were observed at the grain boundaries and gradually became thicker and longer when compared with the solution-treated magnet, which indicated that there was a gradual phase transformation at the grain boundaries.

**Figure 5:**
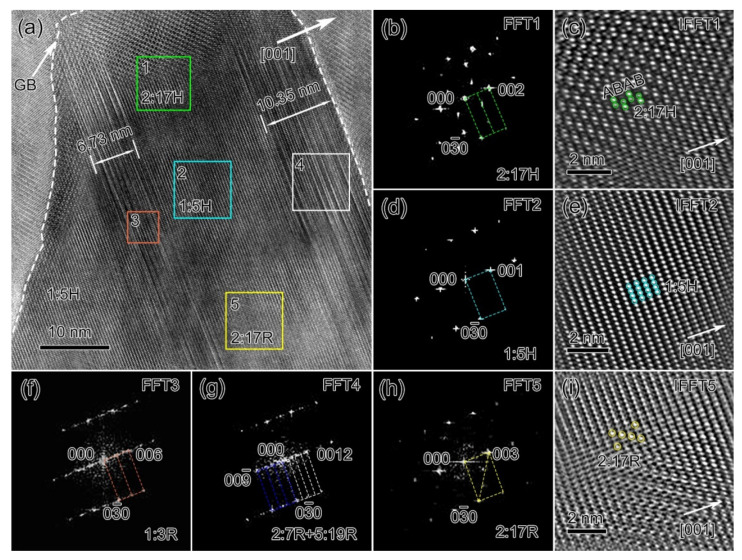
HR-TEM image (**a**), FFT patterns and IFFT images of the 2:17H phase (**b**,**c**), 1:5H precipitates (**d**,**e**), 1:3R precipitates (**f**), Sm_n+1_Co_5n−1_ precipitates (**g**) and the 2:17R phase (**h**,**i**) at the grain boundary for solution-treated Sm_25_Co_46.9_Fe_19.5_Cu_5.6_Zr_3.0_ magnets.

**Figure 6:**
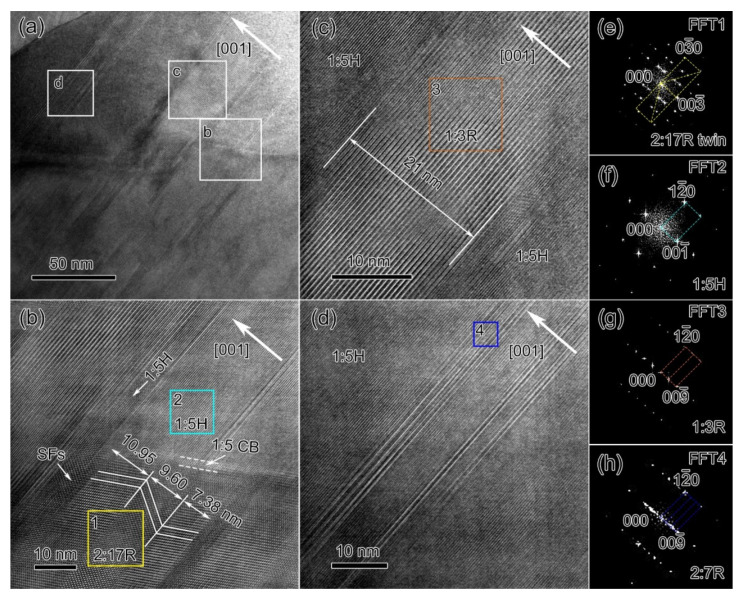
Bright-field image (**a**), HR-TEM images (**b**–**d**), and FFT patterns of 2:17R twin (**e**), 1:5H precipitates (**f**), 1:3R precipitates (**g**), and 2:7R precipitates (**h**) at the grain boundaries for Sm_25_Co_46.9_Fe_19.5_Cu_5.6_Zr_3.0_ magnets aged for 0.5 h at 810 °C.

**Figure 7:**
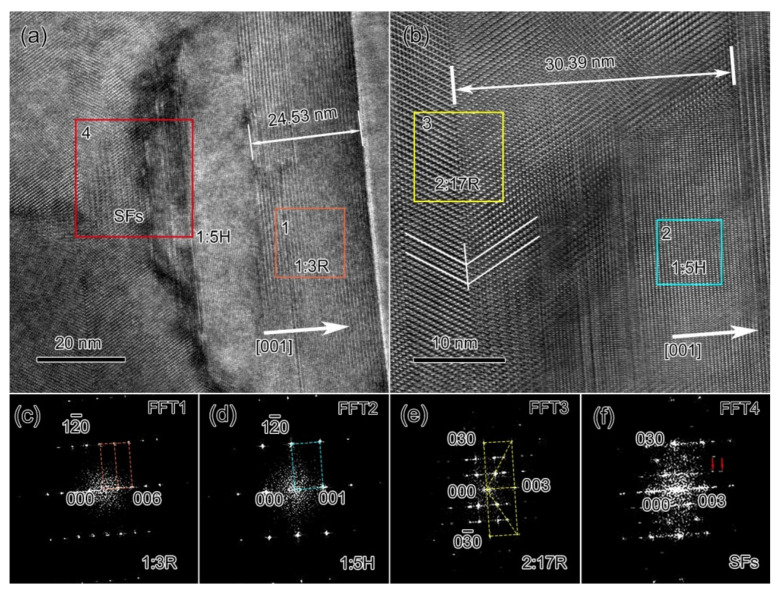
HR-TEM images (**a**,**b**) and FFT patterns of 1:3R precipitates (**c**), 1:5H precipitates (**d**), 2:17R twin (**e**), and SFs (**f**) at the grain boundaries for the final Sm_25_Co_46.9_Fe_19.5_Cu_5.6_Zr_3.0_ magnets.

To reveal the grain boundary microstructure evolution, we then performed detailed HR-TEM characterizations of the regions highlighted by white squares in Figure 3; the results are illustrated in Figure 5, Figure 6 and Figure 7. Figure 5a is the HR-TEM image close to the grain boundary (the white square in Figure 3c) of the solution-treated sample. The fast Fourier transformation (FFT) pattern of the green square region contained {021} and {011} superlattice reflections, revealing that this was the undecomposed 2:17H phase; the inverse fast Fourier transformation (IFFT) image of the 2:17H phase clearly revealed that the Sm atoms with bright contrasts had a typical ABAB double-layer stacking sequence (Figure 5b,c). There was a nanocluster with a brighter contrast in the cyan square region in Figure 5a; the corresponding FFT pattern (Figure 5d) contained no superlattice reflection, revealing that it was the 1:5H precipitate. The corresponding IFFT image showed that the local enrichment of Sm atoms in the bright contrasts had assembled the 1:5H lattice (Figure 5e). Note that the 1:5H precipitate occupied the {001} basal plane instead of the {011}_2:17R_ pyramidal plane, unlike that observed in the grain interiors where the 1:5H phase distributed at the {011}_2:17R_ cell boundary (Figure 4b,c). The ~1/2 {001}*_2:17R_ positions showed a strong intensity in the FFT3 pattern of the orange square region, which was attributed to the Zr-rich 1:3R precipitates (Figure 5f). As shown in Figure 5a, the 1:3R precipitates with a thickness of ~6.73 nm occupied the {001} basal plane. The phase mixture Sm_n+1_Co_5n−1_ nanoprecipitates (~10.35 nm) were judged from the FFT4 pattern (the corresponding *g* vectors are indicated in blue and white dashed lines, respectively). The diffuse streaks along the [001]*_2:17R_ axis suggested they were the phase mixture of 2:7R and 5:19R, which had superlattice reflections at 1/3 and 2/3, 1/4, and 1/2 and 3/4 {003}*_2:17R_ positions, respectively (Figure 5g). In addition, near the grain boundary, the nanosized 2:17R phase was also observed, as demonstrated by the FFT pattern and IFFT image (Figure 5h,i). Consequently, at the solution-treated state, the grain boundary region contained multi-phases, 2:17H, 2:17R, 1:5H, and Sm_n+1_Co_5n−1_. According to the literature [43], 2:17R and 1:5H can be deemed early-stage decomposition products of 2:17H and the Sm_n+1_Co_5n−1_ phases can be deemed primary precipitates formed due to grain boundary segregation during the rapid quenching process. Therefore, the observed 2:17H nanoparticle could be deemed an undecomposed precursor.

Figure 6 shows the TEM characterizations of the microstructure at the grain boundaries of the 0.5 h-aged magnet. A careful live FFT analysis revealed that there was no 2:17H phase in the observed region, indicating the further decomposition of 2:17H into 2:17R and 1:5H. Three regions were selected to perform further HR-TEM characterizations, as indicated by the white squares in Figure 6a. When compared with the solution-treated state, there were noticeable microstructural changes: (i) the 2:17R variants became wider, as shown in Figure 6b when compared with those shown in Figure 4a,b; (ii) the 1:5H precipitate also became larger, which can be clearly seen in Figure 6b,d; (iii) the Sm_n+1_Co_5n−1_ precipitates also grew (as shown in Figure 6c) and the thickness was ~21 nm, much thicker than the ~6.73 nm observed at the solution-treated state (Figure 5a). Note that the 1:5H precipitates were constrained at the grain boundaries, sandwiched between the Sm_n+1_Co_5n−1_ precipitates and the 2:17R nano-variants. As shown in Figure 6c,d, the 1:5H precipitates were even sandwiched between two Sm_n+1_Co_5n−1_ nano-sheets. These results indicated that further decomposition occurred after short-term aging and the 1:5H and Sm_n+1_Co_5n−1_ precipitates grew simultaneously but were mixed.

Figure 7 shows the HR-TEM characterizations at the grain boundaries of the final magnet. In Figure 7a (the larger white square in Figure 3g containing a sharp grain boundary), a ~24.5 nm thick 1:3R precipitate was observed, which was wider than those for the other two magnets. Adjacent to the grain boundary, as shown in Figure 7b (the enlarged view of the smaller white square in Figure 3g), the ~30.39 nm thick 2:17R variant was observed, indicating the further ordering of the 2:17R phase with the extended aging time. The 1:5H precipitate could still be observed, sandwiched between the 2:17R variant and the 1:3R grain boundary precipitate. However, the size of the 1:5H grain boundary precipitate was much smaller than that observed in the 0.5 h-aged magnet. The structures of 1:5H, 1:3R, and 2:17R were justified by the corresponding FFT patterns, as shown in Figure 7c–e. Remanent SFs were also observed near the 2:17R variants, as reflected by the FFT pattern (Figure 7f). When compared with the 0.5 h-aged magnet, the grain boundary of the final magnet contained more Sm_n+1_Co_5n-1_ precipitates and fewer 1:5H precipitates, indicating that the 1:5H precipitates could transform into Sm_n+1_Co_5n−1_ during the aging process.

## 4. Discussion

**Figure 8:**
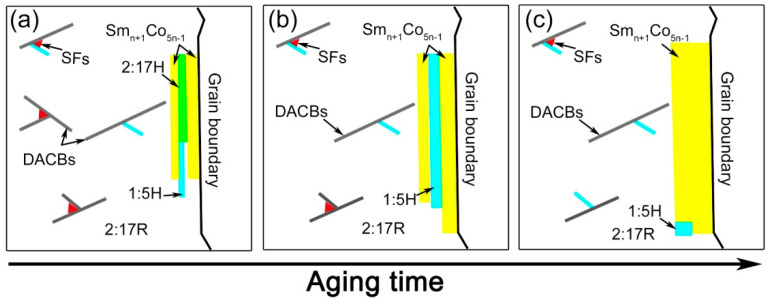
Schematic illustrations of the microstructure of a grain boundary derived from the TEM characterizations for Sm_25_Co_46.9_Fe_19.5_Cu_5.6_Zr_3.0_ magnets. Solution-treated (**a**), aged for 0.5 h at 810 °C (**b**), and the final magnet (**c**).

We schematically illustrate the grain boundary microstructure evolution in Figure 8. The involved phase transformation during the aging process can be summarized as follows.

1. A further decomposition of 2:17H into 2:17R and 1:5H/1:3R occurred at the grain boundaries as also observed within the grain interiors [19].
2. Due to the sparser DACBs, the 1:5H precipitates were constrained between 2:17R and Sm_n+1_Co_5n−1_ primary precipitates, and further transformed into Sm_n+1_Co_5n−1_.

Note that the transformation from 2:17H to 2:17R and the precipitation/growth of the 1:5H phase occurred simultaneously. As 2:17H has been deemed the precursor of 2:17R [20,44], the existence of 2:17R nanotwins and SFs (2:17R’) can be understood in terms of the dislocation glides for the transformation from 2:17H to 2:17R by the glides of a/3 {001}-<120> partial dislocations. The partial dislocation glides were diffusion-controlled. Thus, at the early decomposition stage, the successful nanoscale glides at the local regions will create 2:17R nanotwins whereas the incomplete glides will lead to SFs [20]. The growth of 2:17R variants is at the expense of reducing the basal SFs that are frequently observed at grain boundaries. Grain boundaries enriched with vacancies, providing spaces for the dislocation glide and atomic diffusions, make the recovery and growth of recrystallized cells at the pre-existing grain boundaries easier than that in the grain interiors [45]. Thus, the 2:17R ordering will be higher at the grain boundaries than at the grain interiors after aging. The easier ordering of 2:17R then results in faster kinetics in both the defect dissociation and cell growth. As a result, the nucleation sites of 1:5H precipitates are reduced. Therefore, the decomposition products (both 1:5H and 1:3R precipitates) have to be constrained at the grain boundaries.

The grain boundary evolution is also closely related to the elemental redistributions. Within the grain interiors, the 2:17H to 2:17R + 1:5H + 1:3R transformation also evolves the gradual solute partitioning [2,6] where the 2:17R cells are enriched with Fe/Co and depleted in Sm, the 1:5H precipitates are enriched with Sm/Cu and depleted in Fe/Co, and the 1:3R precipitates are enriched with Zr and have an even higher Cu content than the 2:17R phase, e.g., Figure 6 in [20]. Sm_n+1_Co_5n−1_ precipitates have been frequently observed in solution-treated Fe-rich magnets due to the segregation-related primary precipitation effect, i.e., the local enrichments of Zr and Cu [23,24,25,29,42,43,46]. During the aging process, the decomposition-induced 1:5H and 1:3R precipitates constrained at the grain boundaries are mixed with the Sm_n+1_Co_5n−1_ primary precipitates. Due to elemental redistribution, the Cu-enriched 1:5H precipitates will further transform into the Sm_n+1_Co_5n−1_ phase and the 1:3R precipitates will further thicken the Sm_n+1_Co_5n−__1_ primary precipitates because 1:3R is one of the series of Sm_n+1_Co_5n−1_ compounds. The transformation from 1:5H to Sm_n+1_Co_5n−1_ at the grain boundary is supported by our recent aging temperature-dependent and aging time-dependent studies where the grain boundary Sm_n+1_Co_5n−1_ precipitates become thicker either after aging at higher temperatures or for a longer time [21,24]. For instance, after aging for over 30 h, abnormal cell growth occurred close to the grain boundaries, which could even form micron-sized 2:17R cells. As shown in Figure 6 of [24], both Zr and Cu contents within the grain boundary Sm_n+1_Co_5n−1_ precipitates were higher than the precipitate-free zones (large 2:17R cells). The abnormal cell growth of the gradually thickened Sm_n+1_Co_5n−__1_ phase around the large 2:17R cells could be attributed to the re-dissolution of the Cu-enriched 1:5H and Zr-enriched 1:3R precipitates formed at an early stage.

As the Sm_n+1_Co_5n-1_ precipitates are magnetically softer than the 1:5H cell boundary precipitates [13], it is not surprising that they would be the preferable sites of reverse domain nucleation or the depinning of magnetic domain walls. Due to easy cell growth, the resultant sparser 1:5H cell boundary precipitates will also lead to a weaker pinning strength at the grain boundaries than that at the grain interiors with more 1:5H cell boundary precipitates. We noticed in a very recent work [7] that the fraction of Sm_n+1_Co_5n−1_ primary precipitates can be reduced by optimizing the solution-treatment parameters, e.g., extending the homogenization time at a proper temperature, and this could indeed strengthen the magnetic hardening effect (a better squareness factor and a larger maximum energy product) in Fe-rich Sm-Co-Fe-Cu-Zr magnets after aging. Therefore, a possible approach to minimize the grain boundary deficiencies is to avoid elemental segregation prior to an aging treatment.

## 5. Conclusions

The grain boundary microstructure evolution of a model magnet Sm_25_Co_46.9_Fe_19.5_Cu_5.6_Zr_3.0_ (wt. %) during an aging process was investigated. Unlike the grain interiors, the early-stage 1:5H precipitates at the grain boundaries were constrained between the 2:17R nano-variants and the Sm_n+1_Co_5n−1_ primary precipitates, which could be attributed to easier cell growth (2:17H to 2:17R transformation) that reduces the nucleation sites (pyramidal cell boundaries) of the 1:5H precipitates. At the grain boundaries, the Sm_n+1_Co_5n−1_ precipitates further grew after aging due to the further transformation of 1:5H to Sm_n+1_Co_5n−1_. These results clearly revealed why the grain boundaries are the weaker pinning sites in 2:17-type Sm-Co-based permanent magnets.

## Data Availability

Data sharing is not applicable.
